# PASH syndrome: a novel surgical approach^☆^

**DOI:** 10.1016/j.abd.2020.08.034

**Published:** 2021-11-12

**Authors:** Irina Cawen, Jorge Navarrete, Caroline Agorio

**Affiliations:** a Department of Dermatology, Hospital de Clínicas “Dr. Manuel Quintela”, Montevideo, Uruguay; b Department of Dermatology, Hospital Padre Hurtado, Santiago, Chile

Dear Editor,

PASH syndrome is a rare autoinflammatory syndrome (AIS), which consists of pyoderma gangrenosum (PG), acne conglobata and hidradenitis suppurativa (HS).^1^ No standard treatment has been determined, although case reports have focused on systemic antibiotics, immunosuppressants and biologics.^2^, ^3^

A 45-year-old male presented with a 20-year history of HS on axillae and the inguinal region (Hurley stage III) (Fig. 1A and B). He had diabetes in treatment with metformin, and chronic hepatitis B infection. A sacrococcygeal pilonidal cyst was present (Fig. 1C), as well as ice pick scars (from acne conglobata in his adolescence) (Fig. 1D). Treatment for his HS with multiple systemic antibiotics had failed. Immunosuppressants/biologics were contraindicated due to hepatitis B infection. Wide surgical excision was performed under local anesthesia (axillae and inguinal area, sequentially), with the approximation of borders and secondary intention healing (Fig. 2). The patient was instructed to maintain abduction.

**Figure 1:**
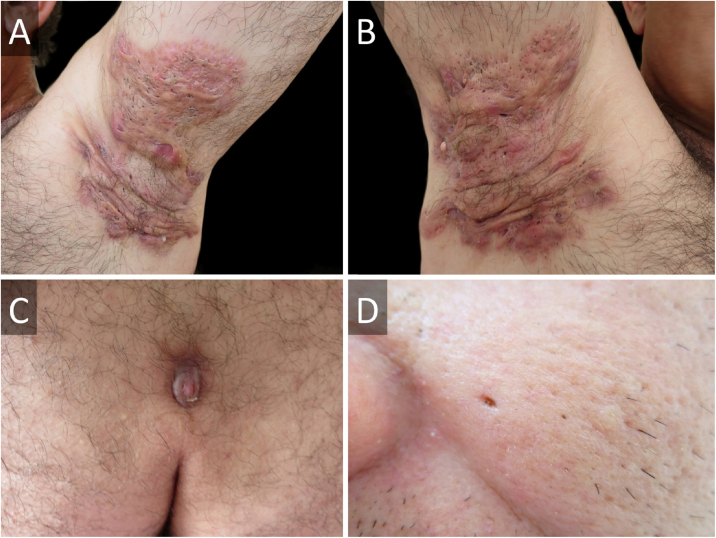
Hidradenitis suppurativa of (A), left and (B), right axillae before surgical intervention. (C), Sacrococcygeal pilonidal cyst. (D), Ice pick scar from severe acne.

**Figure 2:**
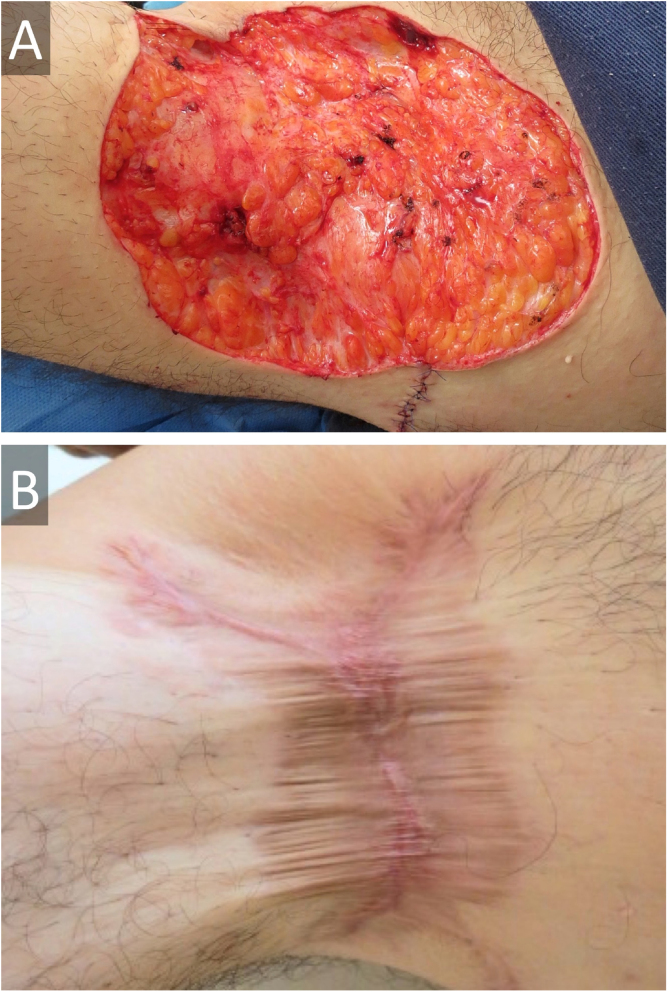
(A), Wide surgical excision of hidradenitis suppurativa on right axilla, with approximation of borders and (B), secondary intention healing with calcium alginate followed by hydrocolloid dressings at 36 weeks, with certain retraction, but no functional impairment.

Six weeks after surgery, he developed a rapidly growing lesion on his left leg (Fig. 3A), with intense pain, but no systemic involvement. This was interpreted as a bacterial skin infection or possibly PG, and empirical intravenous broad-spectrum antibiotics were initiated (penicillin, clindamycin, and ciprofloxacin for ten days), with clobetasol 0.05% ointment. He showed a good response, with cribriform scarring (Fig. 3B). Histopathology showed a dense neutrophilic infiltrate. Cultures were consistently negative. Diagnosis of PG was made, and we were able to categorize our case as PASH syndrome. Lab exams (CBC, ESR, liver and kidney function tests), colonoscopy, and upper endoscopy were normal.

**Figure 3:**
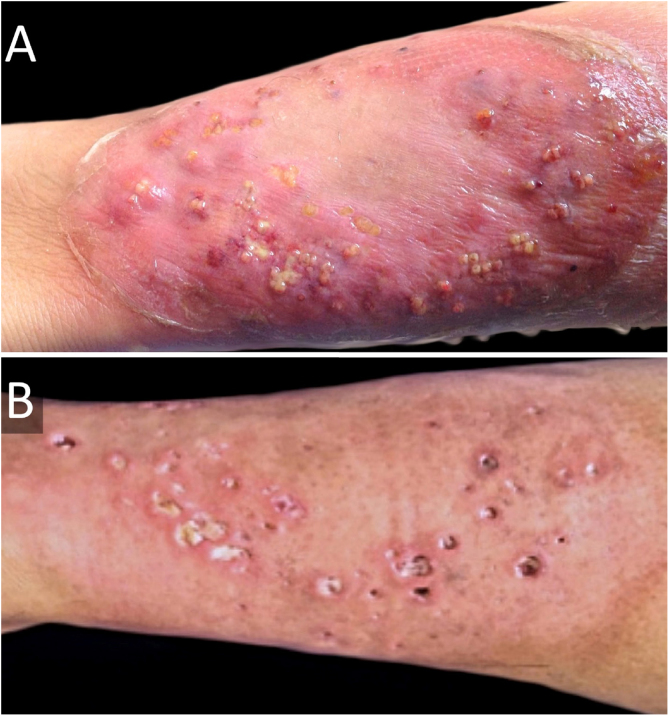
(A), Pyoderma gangrenosum (erythematous lesion with blisters, pustules, and a purplish hue) on his left leg before intravenous broad-spectrum antibiotics and topical high-potency corticosteroids. (B), One month after treatment; cribriform scarring can be seen.

Sequence analysis and deletion/duplication testing of genes LPIN2, MEFV, MVK, NLRP3, PSTPIP-1, and TNFRSF1A showed no pathogenic variants.

With a 36-month follow-up, the patient has had no recurrence of PG or HS.

The diseases that constitute PASH syndrome seem to have independent temporal behaviors and responses to treatment. There are 31 other PASH cases in the available literature, of which 29 have a genetic study, showing heterogenic mutations (previously found in other AIS) in eight cases (8/29; 27.6%).

Pilonidal cysts have not been described in PASH syndrome, although they are part of the follicular occlusion tetrad (along with dissecting cellulitis).

While systemic corticosteroids are the treatment of choice for PG, systemic antibiotics and topical corticosteroids are feasible therapeutic options in diabetic patients.

Given our good experience with surgery on non-PASH HS, as well as in other centers, we chose this strategy, and highlight the good results obtained, something not previously described for PASH syndrome.^4^ This also spares the patient from immunosuppression and other adverse effects of biologics, at a lower cost. The risk of pathergy is a limitation. We recommend three intradermal 20-G needle pricks on the ventral forearm and evaluate at 48 hours in patients with HS and severe acne before surgery is considered since they might represent a case of latent PASH syndrome.^5^

A deeper knowledge of etiopathogenesis may lead to targeted therapeutic strategies, however, this will certainly pose a challenge in a disease as polymorphic as PASH. Treatment must therefore be individualized and considering surgery showed promising results, we believe it merits further research.

## Financial support

The *Fundación Manuel Quintela* helped finance the genetic testing.

## Authors' contributions

Irina Cawen: Approval of the final version of the manuscript; critical literature review; data collection, analysis, and interpretation; effective participation in research orientation; intellectual participation in propaedeutic and/or therapeutic; management of studied cases; manuscript critical review; preparation and writing of the manuscript; statistical analysis; study conception and planning.

Jorge Navarrete: Approval of the final version of the manuscript; critical literature review; data collection, analysis, and interpretation; effective participation in research orientation; intellectual participation in propaedeutic and/or therapeutic; management of studied cases; manuscript critical review; preparation and writing of the manuscript; statistical analysis; study conception and planning.

Caroline Agorio: Approval of the final version of the manuscript; critical literature review; effective participation in research orientation; intellectual participation in propaedeutic and/or therapeutic; management of studied cases; manuscript critical review; study conception and planning.

## Conflicts of interest

None declared.
